# Supplementary material for: Understanding complex multiple sublattice magnetism in double double perovskites
Source: Sci Rep. 2021 Nov 5;11:21764. doi: 10.1038/s41598-021-00959-3 (PMC8571433; doi:10.1038/s41598-021-00959-3)
Supplement: Supplementary file 1 — Supplementary Information. [file 41598_2021_959_MOESM1_ESM.pdf]

Supplementary Information

Understanding Complex Multiple Sublattice Magnetism in Double Double Perovskites

Anita Halder[1,2], Shreya Das[1], Prabuddha Sanyal[3], and Tanusri Saha-Dasgupta[1,*]

[1]*Department of Condensed Matter Physics and Material Sciences,*

S.N. Bose National Centre for Basic Sciences, JD Block,

Sector-III, Salt Lake City, Kolkata 700 106, India

[2] *School of Physics, Trinity College, Dublin, Ireland. and*

[3] *Maulana Abul Kalam Azad University of Technology, Kolkata, India.*

PACS numbers: 75.50.-y,71.20.-b,75.10.Dg

In this supplementary, we provide detailed derivation of the spin Hamiltonian, considering the multiple sublattice double exchange and super-exchange mechanism of magnetism for double double perovskites, $CaMnNi(Co)ReO_6$, and also the finite size effect on the Monte Carlo results for CMCRO compound.

I. SPIN HAMILTONIAN

We can write a 3+1 sublattice Kondo lattice spin-Fermion model for these double double perovskites taking the cue from similar models used earlier for double perovskites [1, 2]:

$$\begin{aligned} H_{DE} &= \epsilon_B \sum_{i\sigma} b^{\dagger}_{i\sigma} b_{i\sigma} + \epsilon_{Mn1} \sum_{i} m^{1\dagger}_{i\sigma} m^{1}_{i\sigma} \\ &+ \epsilon_{Mn2} \sum_{i} m^{2\dagger}_{i\sigma} m^{2}_{i\sigma} + \epsilon_{Re} \sum_{i} r^{\dagger}_{i\sigma} r_{i\sigma} \\ &+ t_{B-Re} \sum_{<ij>} (b^{\dagger}_{i\sigma} r_{j\sigma} + h.c.) \\ &+ t_{Mn1-Re} \sum_{<ij>} (m^{1\dagger}_{i\sigma} r_{j\sigma} + h.c.) \\ &+ t_{Mn2-Re} \sum_{<ij>} (m^{2\dagger}_{i\sigma} r_{j\sigma} + h.c.) \\ &+ J_B \sum_{i \in B} \vec{S}^{B}_{i} \cdot b^{\dagger}_{i\alpha} \vec{\sigma}_{\alpha\beta} b_{i\beta} \\ &+ J_{Mn1} \sum_{i \in A'} \vec{S}^{Mn1}_{i} \cdot m^{1\dagger}_{i\alpha} \vec{\sigma}_{\alpha\beta} m^{1}_{i\beta} \\ &+ J_{Mn2} \sum_{i \in A''} \vec{S}^{Mn2}_{i} \cdot m^{2\dagger}_{i\alpha} \vec{\sigma}_{\alpha\beta} m^{2}_{i\beta} \end{aligned} \tag{1}$$

To this double-exchange part of the Hamiltonian is to be added the superexchange terms H_{SE} arising from the interaction between the core spins:

$$\begin{aligned} H_{SE} = J'_{Mn1-Mn2} \sum_{<ij>} \vec{S}^{Mn1}_{i} \cdot \vec{S}^{Mn2}_{j} \\ + J'_{Mn1-B} \sum_{<ij>} \vec{S}^{Mn1}_{i} \cdot \vec{S}^{B}_{j} \\ + J'_{Mn2-B} \sum_{<ij>} \vec{S}^{Mn2}_{i} \cdot \vec{S}^{B}_{j} \end{aligned} \tag{2}$$

As the Mn(A'), Mn(A'') and B d orbital levels are half-filled (those that are not completely filled), hence all the superexchanges are antiferromagnetic. In the limit of $J_B \to \infty$, $J_{Mn1} \to \infty$, $J_{Mn2} \to \infty$, the b_i, m_i^1 and m_i^2 degrees of freedom become spinless [3, 6]. As a result of this transformation, the hopping terms become:

$$\begin{aligned} H &= t_{B-Re} \sum_{<ij>} \left(sin\frac{\theta_i^B}{2} \tilde{b}_i^\dagger r_{j\uparrow} - cos\frac{\theta_i^B}{2} e^{i\phi_i^B} \tilde{b}_i^\dagger r_{j\downarrow} \right) \\ &+ t_{Mn1-Re} \sum_{<ij>} \left(sin\frac{\theta_i^{Mn1}}{2} \tilde{m}_i^{1\dagger} r_{j\uparrow} - cos\frac{\theta_i^{Mn1}}{2} e^{i\phi_i^{Mn1}} \tilde{m}_i^{1\dagger} r_{j\downarrow} \right) \\ &+ t_{Mn2-Re} \sum_{<ij>} \left(sin\frac{\theta_i^{Mn2}}{2} \tilde{m}_i^{2\dagger} r_{j\uparrow} - cos\frac{\theta_i^{Mn2}}{2} e^{i\phi_i^{Mn2}} \tilde{m}_i^{2\dagger} r_{j\downarrow} \right) \end{aligned} \quad (3)$$

In the following, we drop the tilde on the b, m^1 and m^2 operators (and on the site energies) for ease of representation. Integrating the Re t_{2g} electrons out, and neglecting Berry's phase (assuming coplanar spins for simplicity), the Action becomes:

$$\begin{aligned} S &= \beta \sum_{n,i} \left[(i\omega_n - \epsilon_B) \bar{b}_{in} b_{in} + (i\omega_n - \epsilon_{Mn1}) \overline{m}_{in}^1 m_{in}^1 \right. \\ &+ \left. (i\omega_n - \epsilon_{Mn2}) \overline{m}_{in}^2 m_{in}^2 \right] \\ &- \beta \sum_{n,<<jj'>>} \frac{1}{(i\omega_n - \epsilon_{Re})} \left[t_{B-Re}^2 cos \left(\frac{\theta_j^B - \theta_{j'}^B}{2} \right) \bar{b}_{jn} b_{j'n} \right. \\ &+ t_{Mn1-Re}^2 cos \left(\frac{\theta_j^{Mn1} - \theta_{j'}^{Mn1}}{2} \right) \overline{m}_{jn}^1 m_{j'n}^1 \\ &+ \left. t_{Mn2-Re}^2 cos \left(\frac{\theta_j^{Mn2} - \theta_{j'}^{Mn2}}{2} \right) \overline{m}_{jn}^2 m_{j'n}^2 \right] \\ &- \beta \sum_{n,<jj'>} \frac{1}{i\omega_n - \epsilon_{Re}} \left[t_{Mn1-Re} t_{Mn2-Re} cos \left(\frac{\theta_j^{Mn1} - \theta_j^{Mn2}}{2} \right) \overline{m}_{jn}^1 m_{j'n}^2 \right. \\ &+ t_{B-Re} t_{Mn1-Re} cos \left(\frac{\theta_j^B - \theta_j^{Mn1}}{2} \right) \bar{b}_{jn} m_{j'n}^1 \\ &+ \left. t_{B-Re} t_{Mn2-Re} cos \left(\frac{\theta_j^B - \theta_j^{Mn2}}{2} \right) \bar{b}_{jn} m_{j'n}^2 \right] \\ &- \beta \sum_{n,j} \frac{1}{i\omega_n - \epsilon_{Re}} \left[t_{B-Re}^2 \bar{b}_{jn} b_{jn} \right. \\ &+ t_{Mn1-Re}^2 \overline{m}_{jn}^1 m_{jn}^1 \\ &+ \left. t_{Mn2-Re}^2 \overline{m}_{jn}^2 m_{jn}^2 \right] \end{aligned} \quad (4)$$

where $<< ij >>$ denotes next nearest neighbour terms, $< ij >$ denotes nearest neighbour terms,

while $< j >$ denotes onsite terms. $\sum_n$ denotes sum over Matsubara frequencies. We neglect the next nearest neighbour terms, as they are weaker (over longer distance), and the onsite terms, as they are non-magnetic (spin independent), and consider only the nearest neighbour terms. Then, we calculate $U = -\frac{\partial lnZ}{\partial \beta} = \left\langle -\frac{\partial S}{\partial \beta} \right\rangle$ and get the effective spin model as in Ref [2]:

$$\begin{aligned} H_{DE} &= \sum_{<ij>} D'_{Mn1-Mn2} cos \frac{(\theta_i^{Mn1} - \theta_j^{Mn2})}{2} \\ &+ \sum_{<ij>} D'_{Mn1-B} cos \frac{(\theta_i^{B} - \theta_j^{Mn1})}{2} \\ &+ \sum_{<ij>} D'_{Mn2-B} cos \frac{(\theta_i^{B} - \theta_j^{Mn2})}{2} \end{aligned} \tag{5}$$

where the effective exchanges D'_{ij} are given by (neglecting Berry's phase):

$$D'_{A-B} = \frac{1}{\beta} \sum_n \frac{(2i\omega_n - \epsilon_{Re})}{(i\omega_n - \epsilon_{Re})^2} t_{A-Re} t_{B-Re} (\langle \overline{A}_{in} B_{jn} \rangle + h.c.) \tag{6}$$

where i, j are nearest neighbour site indices.

Thus including the superexchange terms, the full model can be written in terms of spin variables (redefining the D-s and J-s to separate out factors owing to lattice coordination numbers):

$$\begin{aligned} H = 4D_{Mn1-Mn2} &\sum_{<ij> i \in A' j \in A''} \sqrt{\frac{1 + \mathbf{S}_i^{Mn1} \cdot \mathbf{S}_j^{Mn2}}{2}} \\ +8D_{Mn1-B} &\sum_{<ij> i \in A' j \in B} \sqrt{\frac{1 + \mathbf{S}_i^{Mn1} \cdot \mathbf{S}_j^{B}}{2}} \\ +8D_{Mn2-B} &\sum_{<ij> i \in A'' j \in B} \sqrt{\frac{1 + \mathbf{S}_i^{Mn2} \cdot \mathbf{S}_j^{B}}{2}} \\ +4J_{Mn1-Mn2} &\sum_{<ij> i \in A' j \in A''} \mathbf{S}_i^{Mn1} \cdot \mathbf{S}_j^{Mn2} \\ +8J_{Mn1-B} &\sum_{<ij> i \in A' j \in B} \mathbf{S}_i^{Mn1} \cdot \mathbf{S}_j^{B} \\ +8J_{Mn2-B} &\sum_{<ij> i \in A'' j \in B} \mathbf{S}_i^{Mn2} \cdot \mathbf{S}_j^{B} \end{aligned} \tag{7}$$

This is the effective classical spin model which was solved in this manuscript using classical Monte Carlo method, and thereby the magnetic ground states of the two double double perovskites

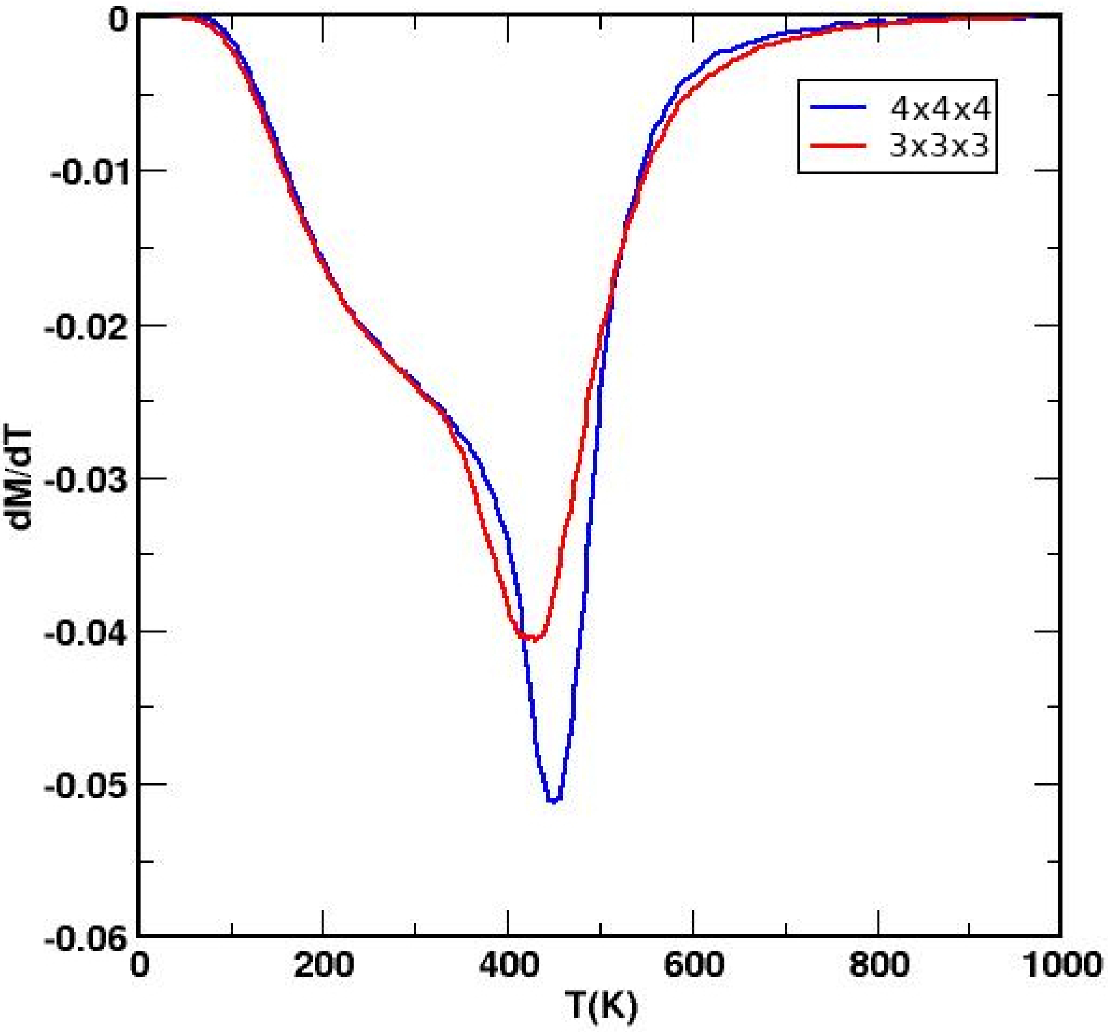

FIG. 1: (Color online) dM/dT curve of CMCRO, obtained by differentiating the M(T) results of Monte Carlo simulations on 3×3×3 and 4×4×4 unit cell simulation boxes.

$CaMnNiReO_6$ and $CaMnCoReO_6$ were obtained. However, this procedure of obtaining an effective magnetic model for double double perovskites is general and can be used for other double double perovskites as well.

II. FINITE SIZE EFFECT

We checked the possible finite size effect in dM/dT curve of CMCRO by repeating the calculations in a large 4×4×4 simulation cell containing 512 atoms. The dM/dT curves in 3×3×3 and 4×4×4 simulation cells for the same choices of J and D values are shown in the Figure 1.

As is seen, the peak and shoulder structure is robust, with the two features, as mentioned in the manuscript arising due to competing effect of Mn1-Co ferromagnetic, and Mn2-Co/Mn1-Mn2 antiferromagnetic interactions. This can be appreciated by comparing the dM/dT curve for Ni compound, which shows a single peak structure with all the magnetic interactions, Mn1-Mn2, Mn1-Ni and Mn2-Ni being of ferromagnetic nature.

III. THERMODYNAMIC STABILITY

In order to ascertain the thermodynamic stability we first calculated the formation energy of the ideal, stoichiometric CMNRO and CMCRO compounds, as well as off-stoichiometric $CaMn_{1.25}Ni_{0.75}ReO_6$ and $CaMn_{0.75}Co_{1.25}ReO_6$ compounds to check their stability with respect to decomposition to elemental fcc Ca, $\alpha-$ Mn, hcp Co/fcc Ni, and O_2 molecule. The calculated values of ideal CMNRO, Mn-rich CMNRO, ideal CMCRO, and Mn-poor CMCRO turned out to be -1.786 eV/atom, -1.824 eV/atom, -1.815 eV/atom and -1.823 eV/atom, respectively. The off-stoichiometric compounds are found to show somewhat higher stability in agreement with report of off stoichiomtry in experimental samples.

We further checked the stability of CMNRO compound, by comparing its formation energy with respect to decomposition to binary compounds like NiO_2, ReO_2, MnO_2, CaO, ReO_3 and ternary compounds like $CaMnO_2$ and Ca_2MnO_4. We considered the following decomposition possibilities.

$$
\begin{aligned}
CaMnNiReO_6 \rightarrow\ & CaMnO_2 + NiO_2 + ReO_2 \\
& CaO + MnO2 + NiO + ReO_2 \\
& CaO + MnO + NiO + ReO_3 \\
& CaMnO_2 + NiO + ReO_3 \\
& (1/2)Ca_2MnO_4 + (1/2)MnO_2 + NiO_2 + ReO_2
\end{aligned}
$$

In all cases $CaMnNiReO_6$ is found to be stablier by 50-100 meV/atom.

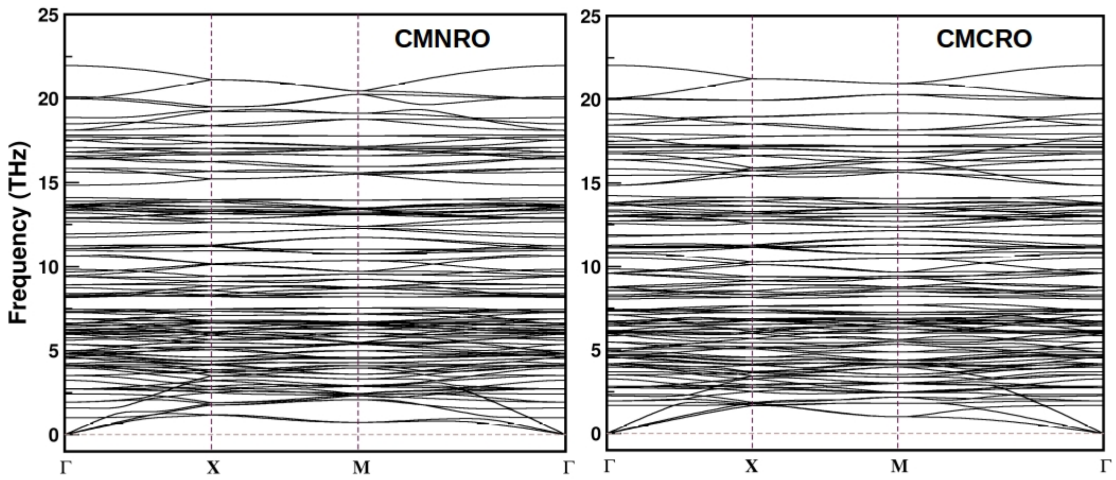

FIG. 2: (Color online) The phonon band structures of CMNRO and CMCRO.

IV. DYNAMICAL STABILITY

We checked the dynamical stability in terms of phonon band structures. The results are shown in the Figure 2. Absence of negative frequencies ascertain the dynamical stability.

---

[1] A. Chattopadhyay and A.J. Millis, Phys. Rev. B **64**, 024424 (2001).

[2] Prabuddha Sanyal and Pinaki Majumdar, Phys. Rev.B **80**, 054411 (2009).

[3] The $J_H \to \infty$ limit of double exchange models was introduced by Anderson and Hasegawa [4] and later studied by P.De Gennes [5] in the context of perovskites. This limit was studied in the context of double perovskites in Ref [6].

[4] P.W. Anderson and H. Hasegawa, Phys. Rev. **100**, 675 (1955).

[5] P. -G de Gennes, Phys. Rev. **118**, 141 (1960).

[6] J.L.Alonso, L.A. Fernandez, F. Guinea, F. Lesmes, and V. Martin-Mayor, Phys. Rev. B **67**, 214423 (2003).
